# Cosmetic and functional results of a newly reconstructed thumb by combining the phalanx of second toe and the great toenail flap transplantation

**DOI:** 10.1186/s13018-020-01986-y

**Published:** 2020-10-06

**Authors:** Yefeng Yin, Xiaomei Tao, Yanzhao Li, Buhe Bao, Ying Ying, Tuya Bao, Jiangning Wang

**Affiliations:** 1 Capital Medical University Affiliated Beijing Shijitan Hospital, 10 Yangfangdian Road, Haidian, Beijing, 100038 People’s Republic of China; 2 Zhongshan Hospital, Dalian University, Liaoning, China; 3 Inner Mongolia University for Nationalities, Tongliao, China; 4 Peking University People’s Hospital, Beijing, China

**Keywords:** Thumb amputation, Toe-to-thumb transfer, Microsurgery

## Abstract

**Background:**

Microsurgical toe-to-hand transfer is a gold standard when it comes to repairing a thumb defect. Great toenail flap, thumbnail valva flap, free great toe, and second toe transplantation are the common methods in thumb reconstruction. Second toe transplantation achieves good function, but poor esthetics. Great toe transplantation achieves better esthetics, but hindered walking, due to the foot’s loss of the great toe and moreover suboptimal thumb function. It is difficult to maintain both functional and esthetic satisfaction in thumb reconstruction.

**Methods:**

We experimented with three different methods of toe to hand transfer. From October 2009 to July 2019, 30 patients with traumatic thumb defects received one of 3 different kinds of thumb reconstruction in our clinic according to their level of amputation. Divided evenly into three groups of ten, group one received a great toe transplantation, group two received a second toe transplantation, and group three received a combined great toenail flap and second toe phalanx transplantation. Each of the patients’ thumbs had different levels of amputation at the metatarsophalangeal joint (MPJ) or distal interphalangeal joint (DIPJ).

**Results:**

One patient suffered from a partial flap necrosis and received a groin flap to cover the defect. No other thumbs had any complications. The functional and esthetic results of both the donor and the recipient sites were satisfactory. Results show that, for patients with traumatic thumb defects, the combined transfer of flap and second toe phalanx was the best option.

**Conclusions:**

Compared to the great toe or second toe transfer, combined free transfer of the great toenail flap and second toe phalanx achieved a substantially better functional and esthetic result in the thumb reconstruction.

## Introduction

Thumb amputation immensely handicaps a hand’s function. Plastic surgeons do their best to construct functional, while at the same time, esthetically pleasing thumbs for their patients. The free toe-to-thumb transfer is a milestone in the clinical application of microsurgery technique [1, 2]. The skin and structure of the toe are very similar to the hand; thus, the functional restoration is an excellent technique that results in minimal morbidity at the donor site [3]. The great toe transfer is recognized as being able to achieve both esthetic appearance and good function in the reconstructed thumb, while the second toe transfer is thought to result in less donor site morbidity [4]**.** The current specific considerations regarding the selection of the toe(s) are greatly based on the surgeon’s experience and patient’s need [5].

It is important to harvest the proper free toe flap by taking the particular thumb defect into account. In this study, we present our new technique of using a free flap combined with the great toenail and second toe phalanx, in addition to necessary vessels, nerves, and tendons, to reconstruct the thumb. We have achieved both better functional and esthetic results when compared to solely great toe or second toe transfers.

## Materials and methods

### Patient data

Thirty patients, who sustained thumb amputations between October 2009 and July 2019, were reviewed in this study. There were 18 male and 12 female patients that were an average of 31 years old (range, 14–48 years). The reasons for amputation were from accidents with manufacturing tools such as: the machine press (10 patients), power saw (6 patients), iron press (6 patients), and drill (8 patients). Twenty patients underwent right thumb amputations and 10 patients had left thumb amputations. Twenty-nine patients received thumb reconstructions immediately after their injuries and 1 patient received a delayed reconstruction 16 years after the accident.

### Surgical procedure

Debriding of the necrosis tissue was first performed in the operation room and the arteries, veins, nerves, and tendons of the injured thumb were identified and isolated. The surgical plan was made according to the size and shape of the defect. The patient’s lifestyle and local condition of the donor site were also considered in the selection of the donor site.

The reconstruction method was chosen based on the thumb amputation level and the patient’s career background. For the defects that involved the distal interphalangeal joint (no matter transverse, oblique, or longitudinal defect), great toe to hand transfers were performed for thumb reconstruction as reported [6–8]. The second toe is smaller and shorter than the normal thumb in appearance, but can achieve better function than the great toe [9, 10]. We used the second toe transfer for patients who suffered metacarpal bone or proximal phalanx defects with higher demands for function and lower demands for appearance. For those who had higher demands for both appearance and function of the reconstructed thumb, the second toe phalanx covered with the great toenail flap was used. Once the operation method was determined, Doppler was used to locate the donor and recipient vessels.

#### Donor site preparation

Incisions were made according to the design (Fig. 7a, b). Dissection was performed to isolate the first and second branches of the dorsal vein and achieve sufficient pedicle length. We then ligated the other vein branches and made incisions at the web and plantar areas to find the first plantar metatarsal artery, the digital nerve, and its branches. We had to next confirm the toe web vascular type and isolate the blood vessels and nerves. If the dorsalis pedis artery was chosen for the anastomosis, isolation of the dorsal artery was performed and the deep plantar branches were ligated to achieve the blood supply model as the dorsal artery-the first dorsal artery-toe artery. Sometimes, the first dorsal metatarsal artery has variation. The dorsalis pedis artery-plantar artery-toe arterial blood supply mode should be maintained for type III dorsal metatarsal arteries. If the first plantar artery was chosen to perform the anastomosis, it would not have been necessary to consider the toe web vascular type or isolated the first dorsal metatarsal artery. The neurovascular bundle was then exposed and dissected together with the free flap. Ligation of the hallux cross artery was carefully performed and the second phalanx was cut according to the defect of the recipient site. This was followed by the isolation of the hallux and 2 phalanx flap.

##### Bone reconstruction

We used wires and Kirschner to fix the bones for earlier functional rehabilitation.

##### Tendon reconstruction

After the second toe phalanx with vessels, nerves, and tendons were fixed to the phalangeal bone of the thumb with Kirschner wire, the extensor tendon and the flexor tendons were repaired and put in their rest positions. If the original amputation included the proximal site of the metacarpophalangeal joint, the pollicis brevis tendon also needed to be repaired. To do accomplish this, two functions needed to be restored. The digital flexion function was restored by replacing the hallucis longus flexor and extensor tendons with the second inherent extensor and the 4th superficial flexor tendons. The function of opposition was achieved by using a dynamic tendon, which provides power to oppose the thumb, as a substitute in a similar way.

##### Nerve reconstruction

Nerves needed to be sutured under tension-free circumstances. The proximal nerve avulsion defect was reconstructed by suturing the second digital nerve (ulnar side) with the donor nerve.

##### Vascular reconstruction

Anastomosis was done to complete the vascular reconstruction. Patients received end to end anastomosis through subcutaneous tunnels between the first dorsal metatarsal artery and the radial artery at the snuffbox, in addition to between the saphenous vein and the deep branch of the cephalic vein. If the diameter of the first dorsal metatarsal artery was too small or absent, anastomosis of the first plantar metatarsal artery and the finger inherent artery was done instead.

##### Donor site closure

The second phalanx flap was then transplanted to the tibial side of the hallux and the exposed bone was covered with the hallux nail and skin flap (it was important to keep the pedicle in tension-free circumstances). The hallux flap was sutured to the skin on the second phalanx flap (Fig. 4a). For the distal thumb joint reconstruction, a full-thickness skin graft on the great toe and direct closure of the second toe was performed; the second toenail flap was used to cover the great toe defect in other patients.

**Fig. 1:**
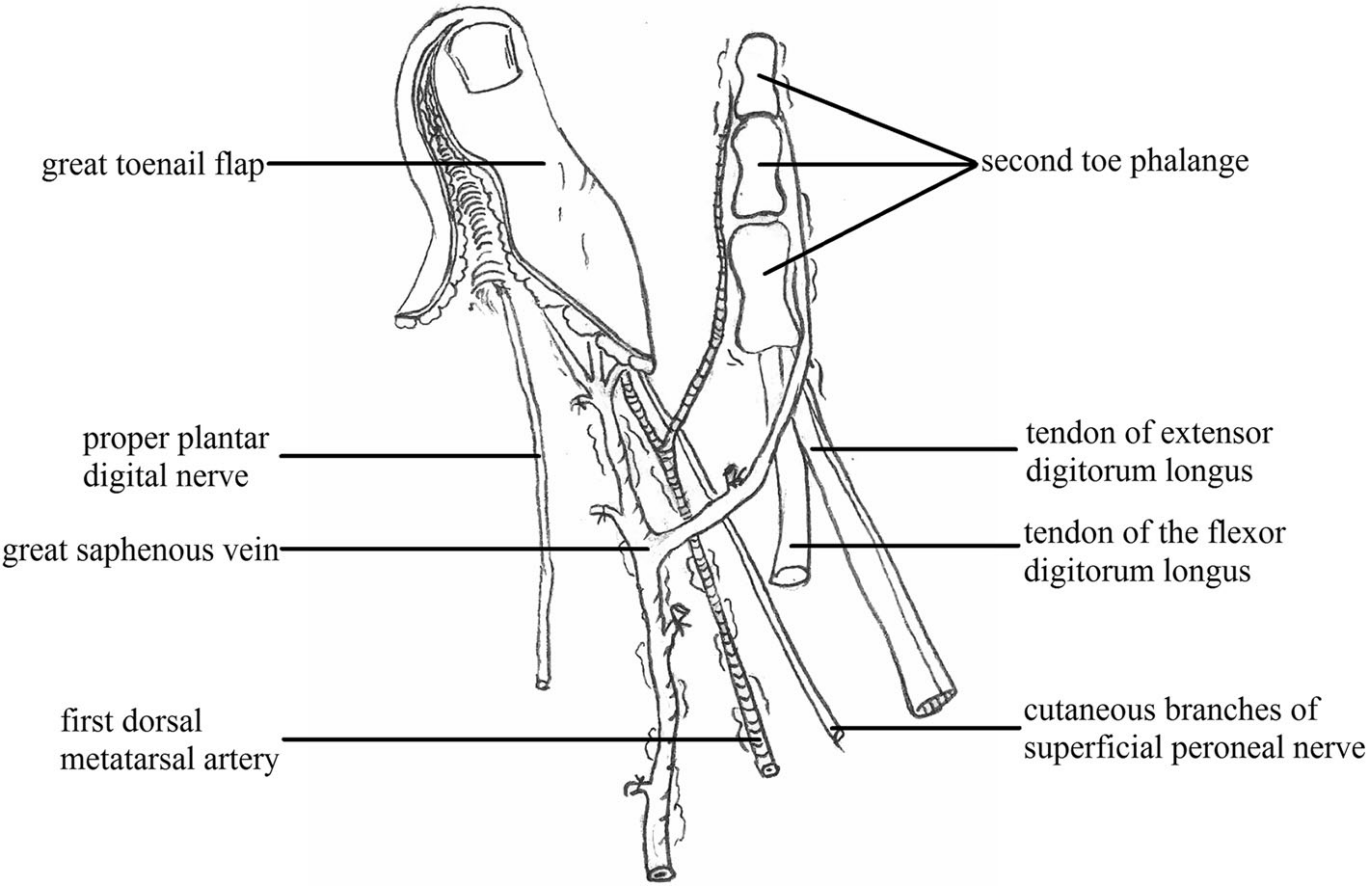
The harvested great toenail flap and second toe phalangeal

**Fig. 2:**
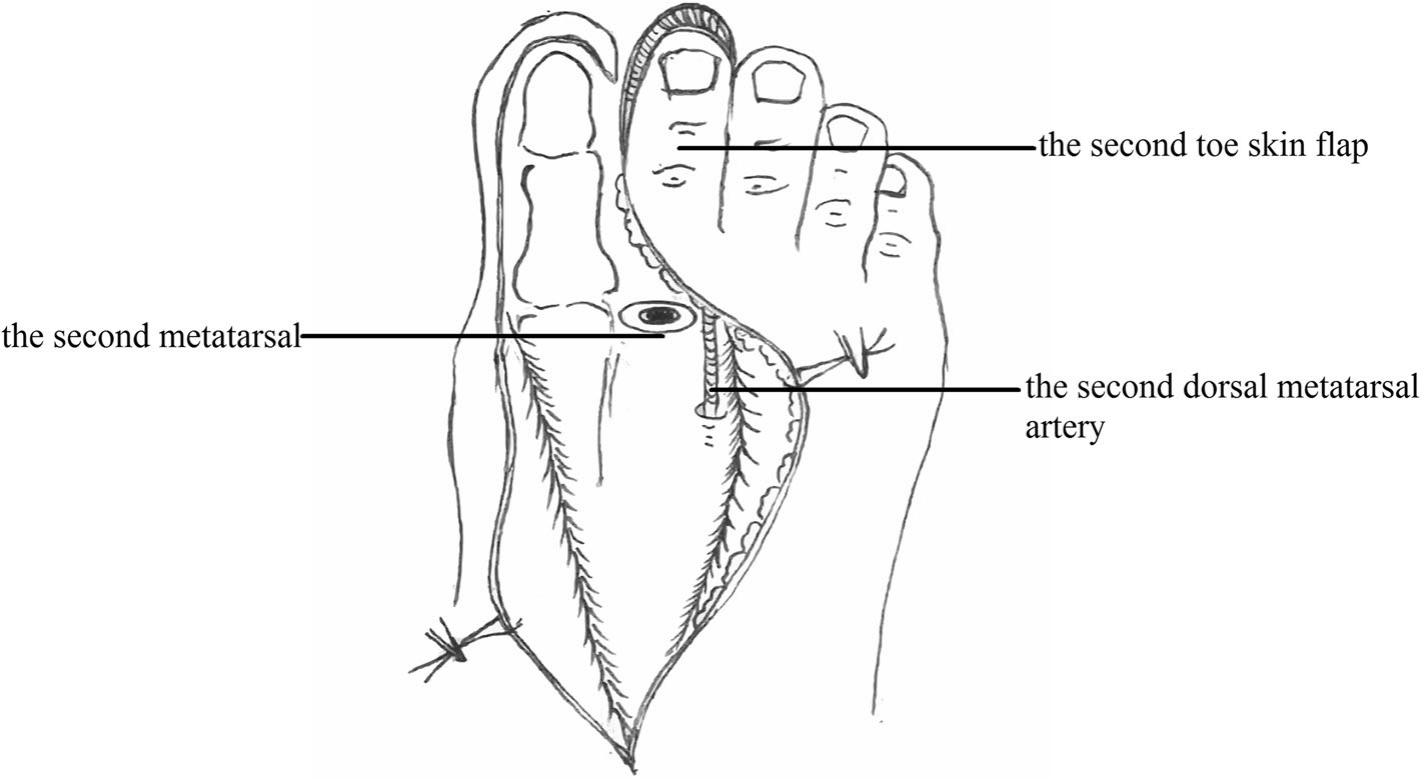
The donor site after the flap was harvested

**Fig. 3:**
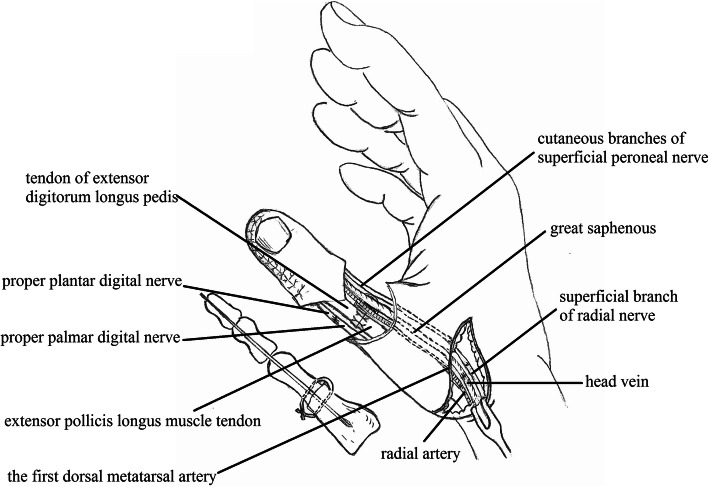
The inset great toenail flap and second toe phalangeal

**Fig. 4:**
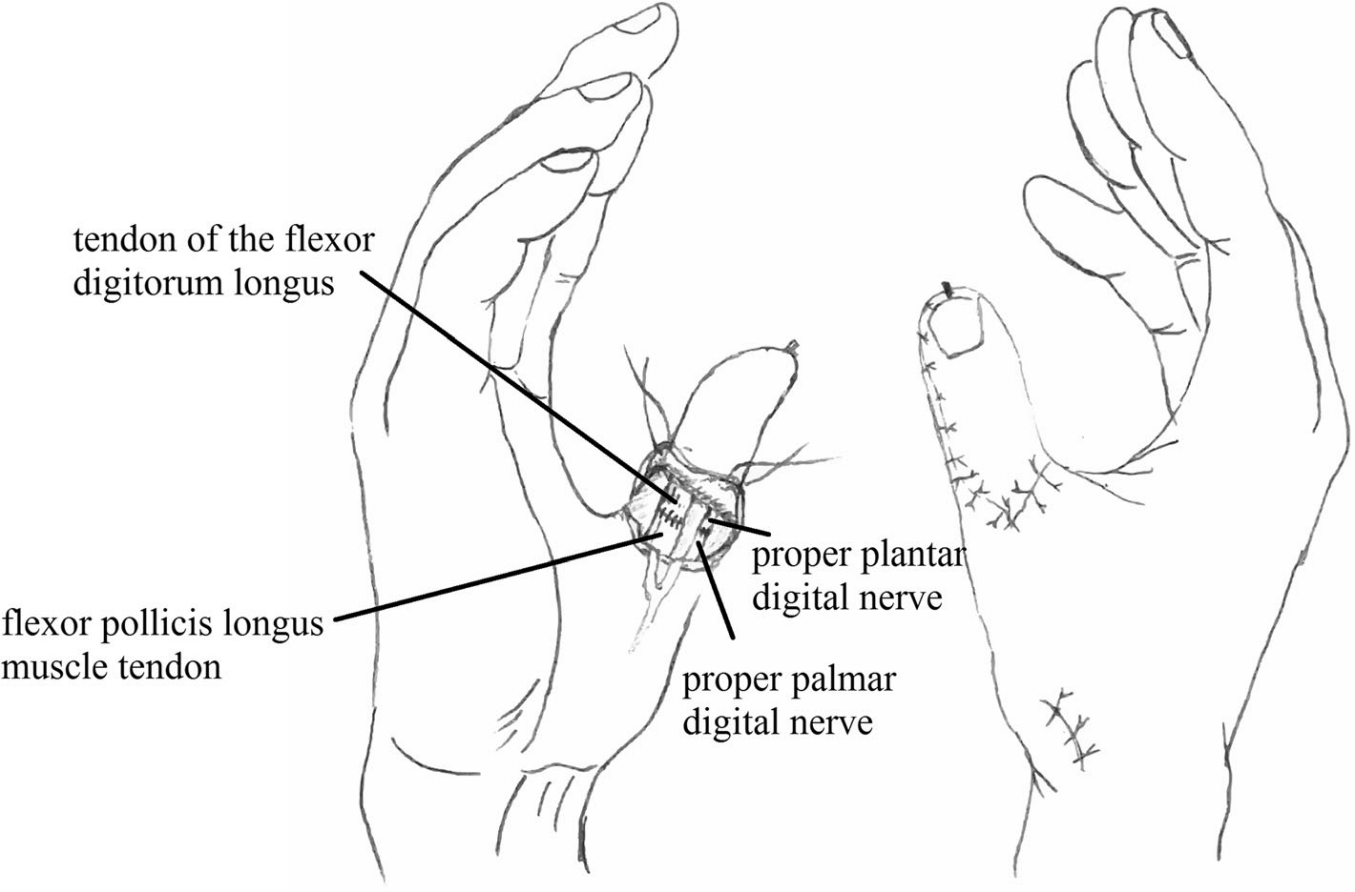
The sutured nerve and tendon of the metacarpal thumb

**Fig. 5:**
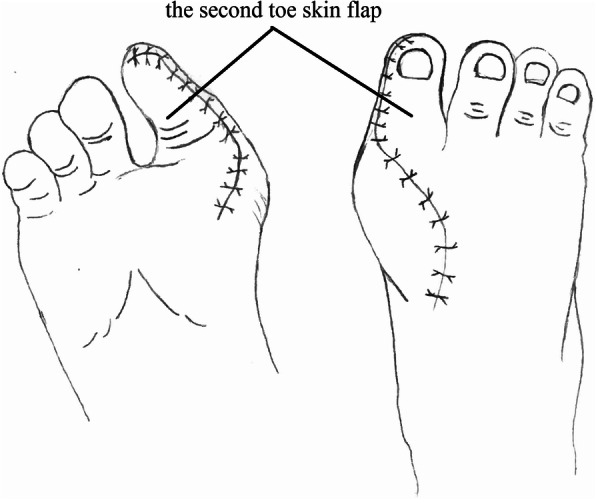
The covering of the defect of the great toe by using the second toe skin flap

The defect of the great toe was able to be easily covered by the second toe skin flap and thus the wound required no necessary skin grafts. Figures 1, 2, 3, 4, and 5 show the surgical procedures and techniques.

Intravenous injection of cefazolin (30 mg/kg) was given prior to surgery and for 1 day postoperatively. If the patient is allergic to cefazolin, intravenous clindamycin (0.6 g, bid) was given instead. Papaverine hydrochloride (30 mg, intramuscular, tid) was given to relieve vasospasm for 10 days. Anticoagulation was achieved with low molecular weight heparin (LMWH). All patients were subcutaneously injected with LMWH (3800 IU/day) after the surgery for 12 days. Normal daily activity of the foot started at 2 weeks postoperation and rehabilitation of the reconstructed thumb started at 4 weeks postoperation.

The Kirschner wire was removed at 6 weeks postoperation after X-ray confirmation of bone healing. Distal interphalangeal joint fusion of the second toe was achieved as planned. Bone fusion of the second toe shortened the length of the reconstructed thumb to a final, close-to-normal size.

*Rehabilitation*. (1) X-rays were taken 6 weeks postoperation to confirm the bone fusion and that the external fixation was removed. Passive and active functional exercises were also started at this time with a hand brace to avoid detrimental flexor and extensor tendon adhesions. (2) Electromagnetic waves were used at the reconstructed thumb pulp to stimulate nerve regeneration. (3) Iron therapy balls and 15 kg rubber bands were used to practice the thumb grip strength, pinch strength, and other muscle functions.

*Rehabilitation schedule*. (1) Postoperative tendon adhesion is the main factor that negatively affects the reconstructed thumb function, but early functional rehabilitation minimizes this problem. The preparation stage starts 4-6 weeks postoperation. In addition to passive movements for 5-10 min, 2 times a day, active movements were practiced with the guidance of a physician. (2) Continuous passive joint motion exercises were started 6 weeks postoperation and were done for 4 weeks. Initial activity was measured with a Baltimore Therapeutic Equipment (BTE, an interactive rehabilitative system) Primus hand function rehabilitation assessment instrument. A total of 5-10b traction strength was increased daily when there was no significant pain during the 30-min exercise. Tendon release was performed if tendon adhesion occurred. (3) At 8-12 weeks postoperation, the muscle strength training stage was begun. Isokinetic training was performed at 60 b/s, 90 b/s, and 120 b/s, 30 times a day. (4) Ten to fourteen weeks postoperation was the hand coordination training stage. According to the work characteristics of the patients, individually simulated vocational training was performed 30-60 min/day. Reassessment of the hand function was performed 6 months postoperation. By individually simulating the patients with vocational training, we were able to prepare the patient to work once again.

Twenty-nine reconstructed thumbs recovered smoothly with no complications. Although 1 case suffered partial flap necrosis, we used the abdominal flap as a lifeboat and the patient recovered successfully. There were no infections at either the donor or recipient sites. Donor sites were evaluated using three domains: foot pain, foot function, and foot ulcers. No patients had complications at the donor site. The average length of the hospital stay in groups I, II, and III was 18.5 days (range, 15–31 days), 17.2 days (range, 14–23 days), and 17 days (range, 14–21 days) respectively. The mean follow-up time was 12 months (range, 8–20 months) in group I, 12 months (range, 7–18 months) in group II, and 12 months (range, 5–19 months) in group III.

## Result

In this case series, 30 patients were separated into 3 groups according to the type of toe-to-thumb operation they received. Group I had 10 cases of great toe transplantations, group II had 10 cases of second toe transplantations, and group III had 10 cases of combined great toenail flap second toe phalanges. Total active motion (TAM) of the transplanted thumbs in group I, II, and III was 68 ± 7.8, 83 ± 5.1, and 83 ± 5.5, respectively, which was compared with the contralateral healthy thumbs of the patients. Sensation recovery was measured with the static 2-point discrimination test (S-2PD). Results of groups I, II, and III were 7.6 ± 1.4 mm, 8.1 ± 1.2 mm, and 7.5 ± 1.3 mm respectively. No patients suffered any painful paraesthesias. The Michigan Hand Questionnaire (MHQ) was used to measure the reconstructed thumb function 10 months after the operation. The subjective self-assessment function and cosmetic scores of the newly replanted thumb are described in Table 1.

**Table 1:**
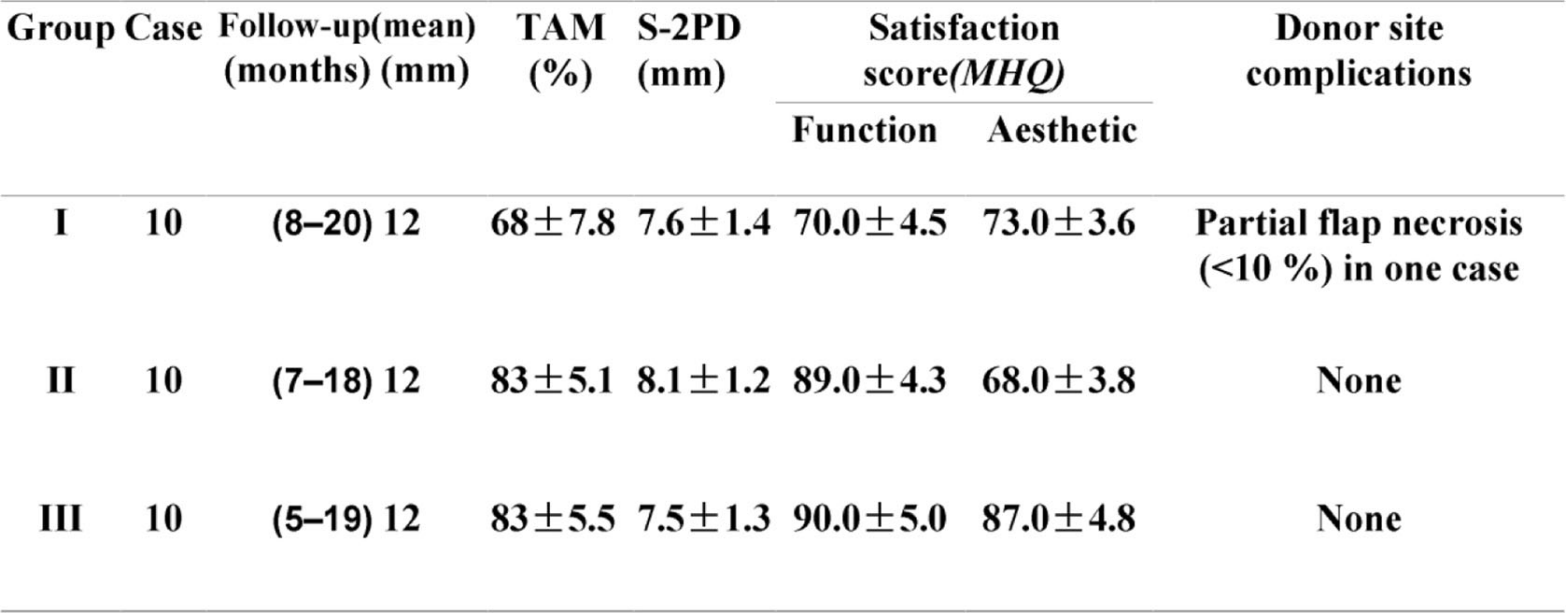
Postoperative results of the new thumbs and complications

*TAM* total active motion (motion recovery evaluated by total active motion compared with the contralateral thumb); *S-2PD* static 2-point discrimination; *MHQ* Michigan Hand Questionnaire

The mean follow-up time was 12 months (range, 8–20 months; SD = 3.47) in group I, 12 months (range, 7–18 months; SD = 2.81) in group II, and 12 months (range, 5–19 months; SD = 3.13) in group III

Group I, thumb reconstruction by great toe transplantation

Group II, thumb reconstruction by second toe transplantation

Group III, thumb reconstruction by the great toe flap and the phalanges of the second toe

### Example case

**Fig. 6:**
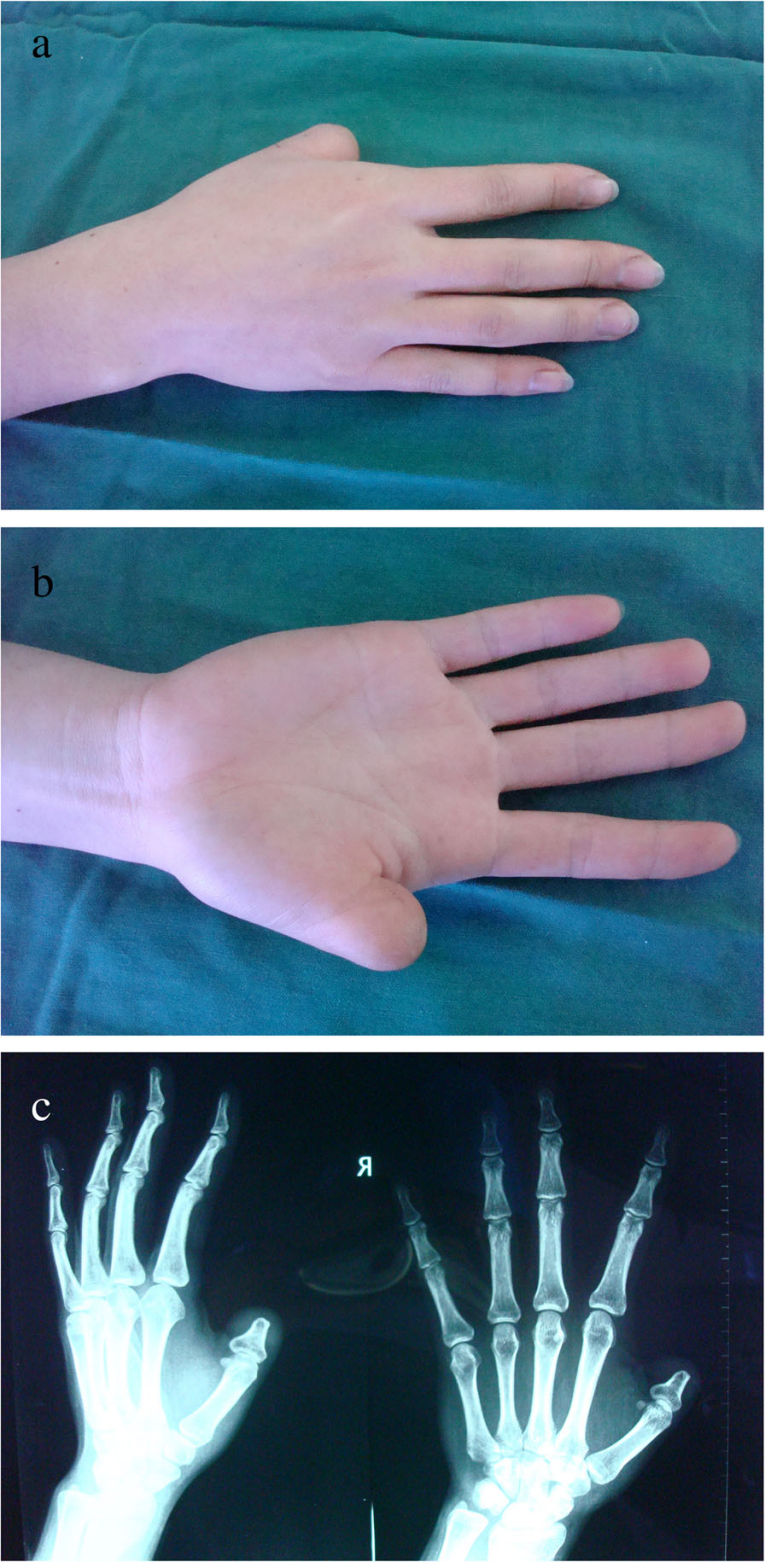
**a**, **b** Preoperative view of the right thumb defects distal to the interphalangeal (IP) joint. **c** Preoperative X-ray of the right hand

**Fig. 7:**
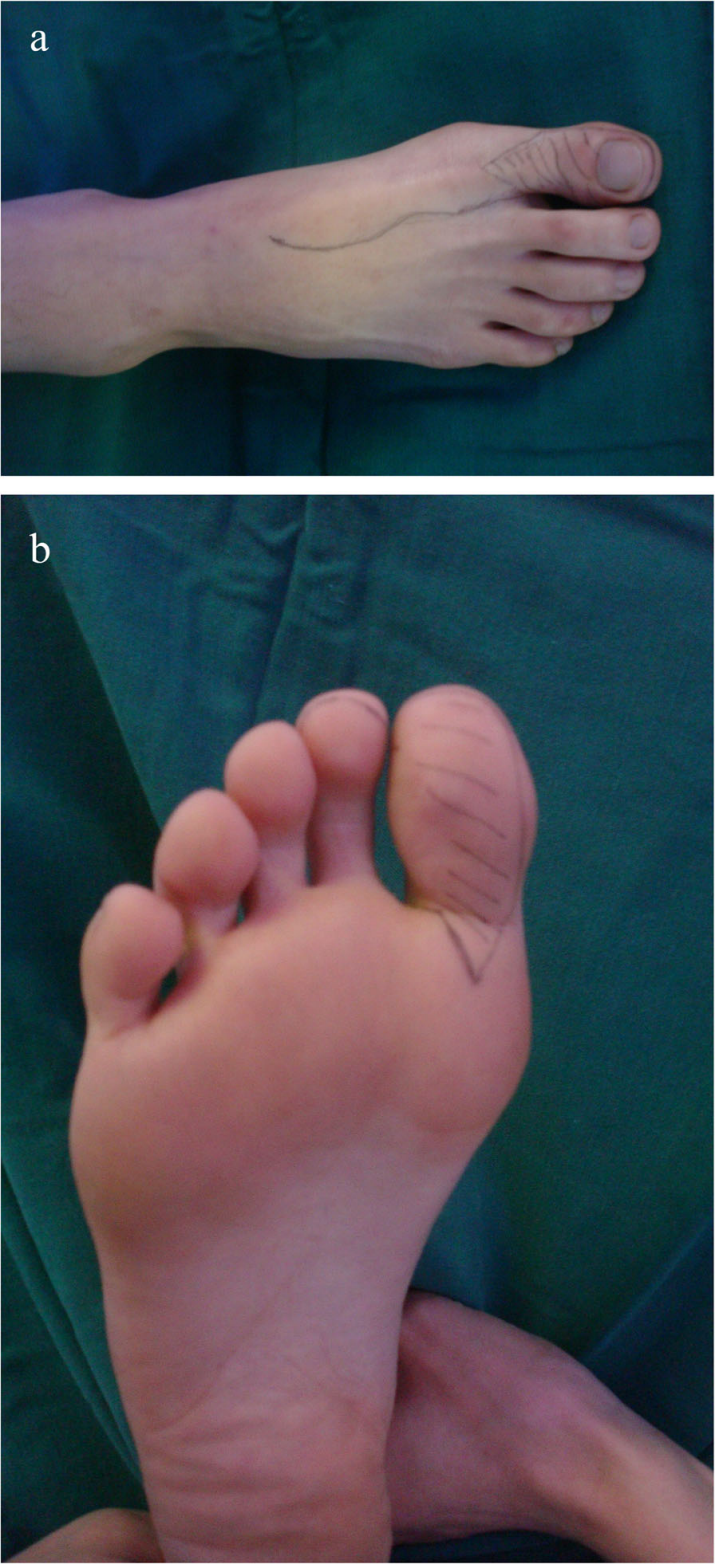
**a**, **b** Operative design

**Fig. 8:**
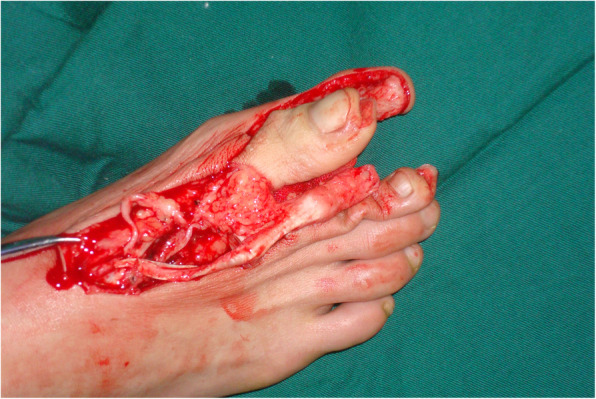
Intraoperation of the great toe flap and the metatarsophalangeal joint of the second toe

**Fig. 9:**
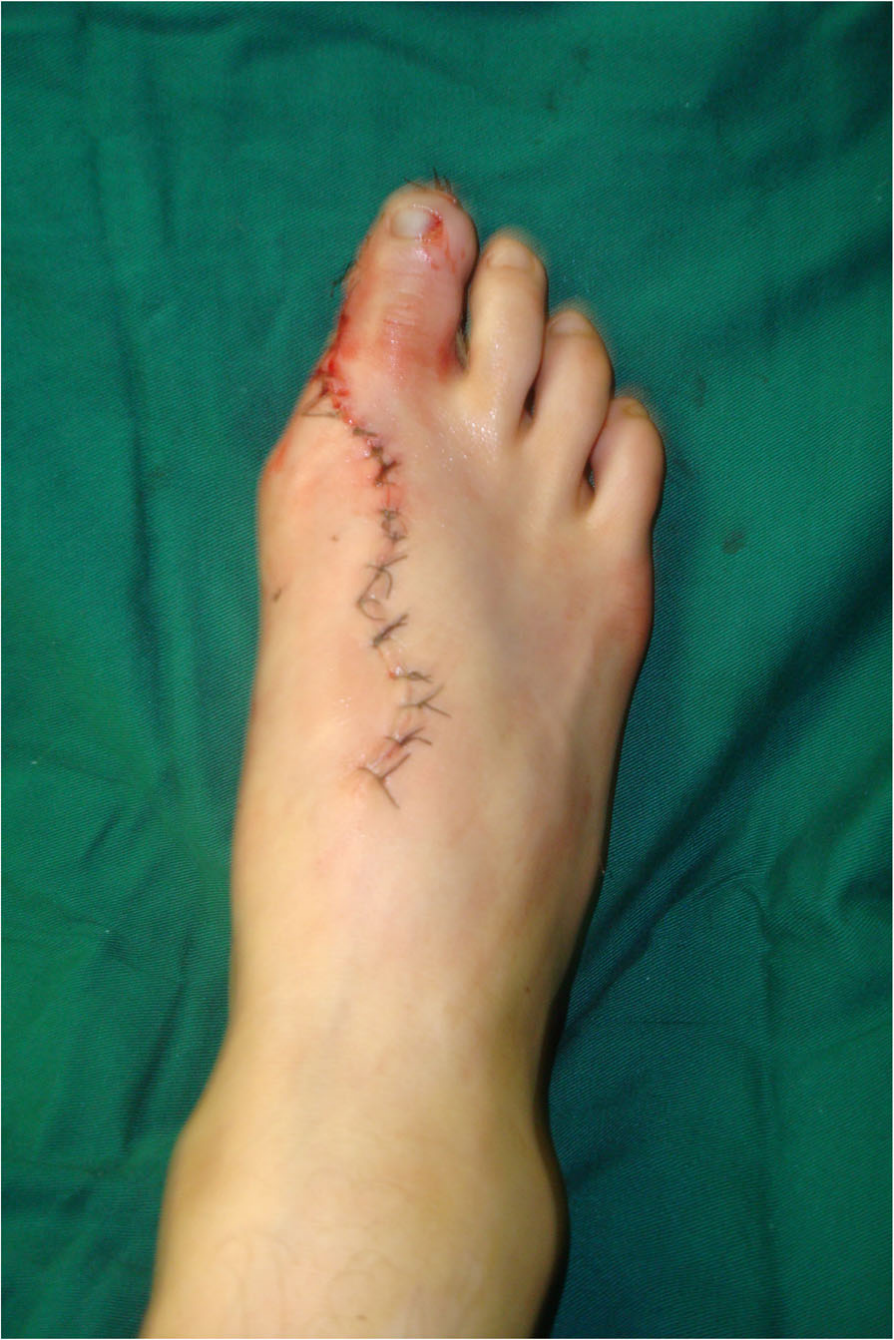
The donor site closed by the second toenail flap

**Fig. 10:**
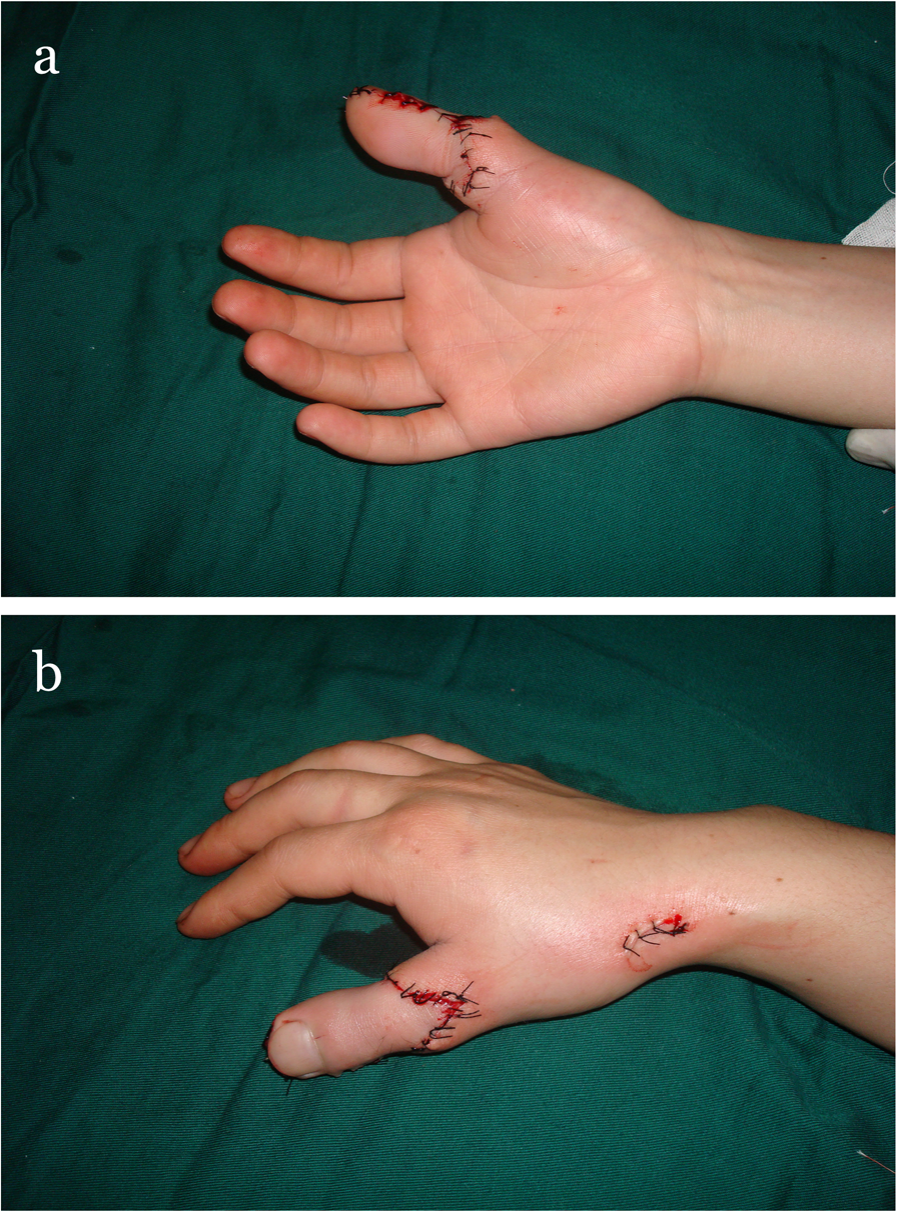
**a**, **b** Postoperative view of the reconstructed thumb and donor site

**Fig. 11:**
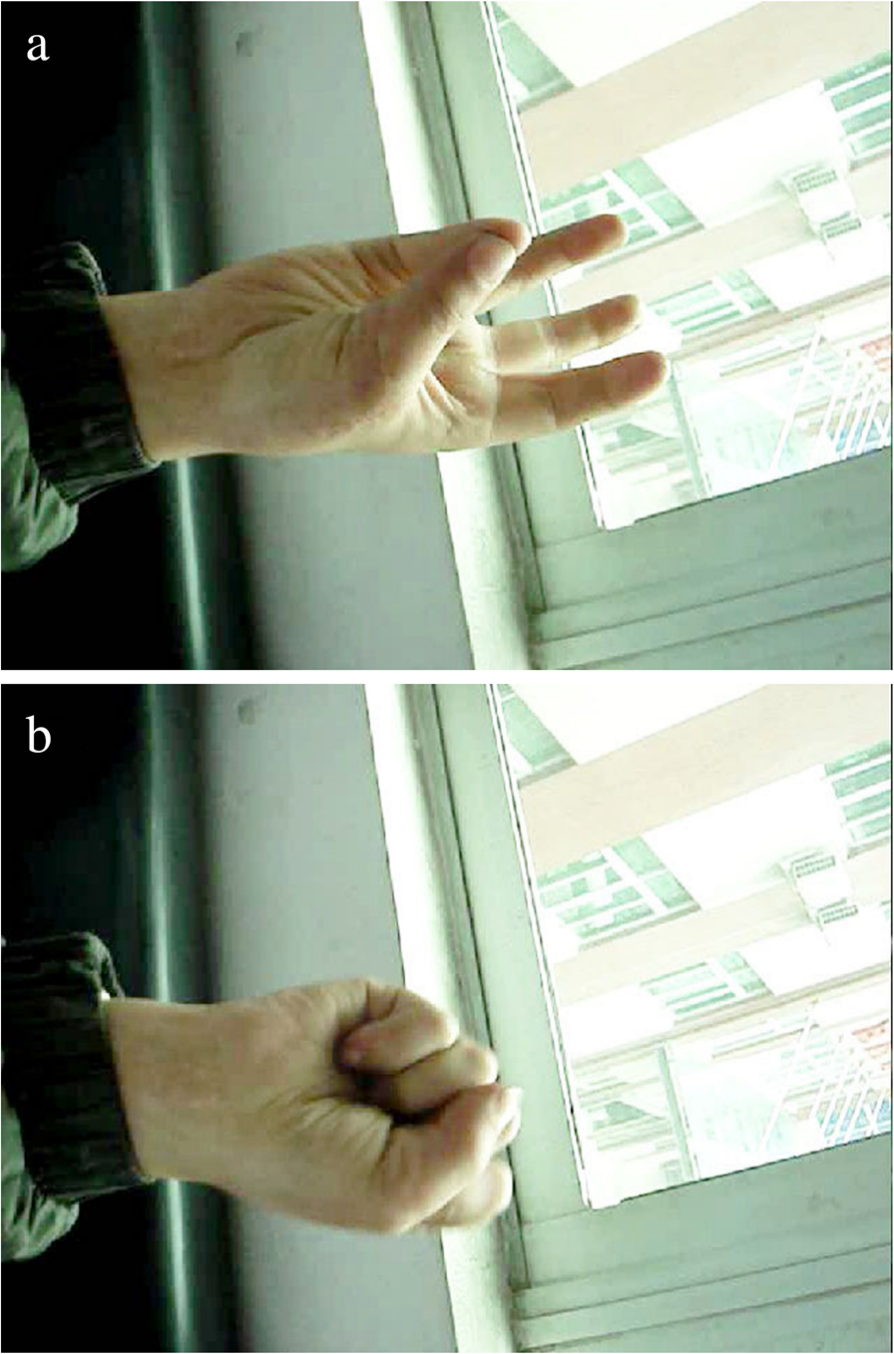
**a**, **b** 6 months of follow-up after operation

A 26-year-old female suffered right thumb amputation at the IP joint 16 years ago (Fig. 6a, b). In order to restore the function and appearance of the affected thumb, toe-to-thumb transplantation was performed. Right hand X-rays showed the defect level to be distal to the metacarpophalangeal joint (Fig. 6c). After a detailed preoperative design (Fig. 7a, b), we harvested the combined flap from the great toenail and metacarpophalangeal joint from the second toe (Fig. 8). The length of the vascular pedicle was 11 cm. The donor site was primarily closed by means of covering the great toe phalangeal with the second toenail flap (Fig. 9). No vein graft or skin graft was performed and a functional thumb with good appearance was achieved (Fig. 10a, b). Good functional and cosmetic result can be achieved by operative treatment (Fig. 11a, b)

## Discussion

The thumb is the most vital part of hand function. The ideal thumb reconstruction from a microsurgical toe transfer should meet the requirements of both form and function with minor donor site morbidity [11–13]. Currently, there are four main types of free toe transfers (total great toe, second toe, great toe wrap around, and trimmed great toe) [8, 12, 14–17]. Using the great toe to hand transfer, the reconstructed thumb gains good nerve recovery and skin texture, but the cosmetic result of the recipient site and the function of the donor site are not satisfactory. The second toe to hand transfer results in good hand function with less donor site morbidity, but it also leads to poor hand esthetics [8, 18].

This study aimed to find a combined technique to achieve better results than the other four primary reconstruction methods. In this study, the great toenail flap and second toe phalangeal were chosen as donors to meet the demands. The combination of the two techniques achieved both good appearance and function. Indications of this combined technique include (1) congenital or acquired thumb absence at any level with non-injured great and second toes; (2) the patient’s preference and agreement for the toe to hand operation method; (3) no present medical history with diabetes, high blood pressure, or cardiovascular or psychological disease; (4) proper local tissue conditions for thumb reconstruction in both the donor site and recipient site.

The main advantages of this combined method are as follows: (1) It combines the advantages of both the great toe and the second toe transfer to achieve an optimal functional and cosmetic result; (2) there is very little handicap to the weight bearing of the donor foot due to the preservation of the great toe phalanx; (3) it results in better appearance of the donor site than a full-thickness skin graft; (4) it is easy to locate the pedicle flap.

Still, the technique also has some disadvantages: (1) There is a total loss of the second toe at the donor site; (2) there is increased difficulty of harvesting the combined flap instead of just the normal toe flap; (3) the operation is time consuming. The contraindications of this combined flap include (1) poor general condition; (2) donor or recipient site infection; (3) previous injury of the great or second toe; (4) social or psychological resistance to toe-to-hand transfer.

There are many factors that should be considered before an appropriate surgical plan is made, such as patient’s age, gender, profession, level of amputation, and hand dominance [19, 20]. In this case series, all the patients that received combined free transfer of the great toenail flap and second toe phalanx achieved not only good function and appearance at their donor sites but also acceptable functional and cosmetic results of the newly reconstructed thumb. The composite transplantation is a great alternative choice for thumb reconstruction.

In this report, 30 cases with thumb amputations were successfully reconstructed with three different methods. Although the technique has some potential risks, our results indicate that the combined free transfer of great toenail flap and second toe phalanx achieves both functional and esthetic satisfaction in thumb reconstruction.

## Data Availability

Not applicable.
